# The effect of the capitation policy withdrawal on maternal health service provision in Ashanti Region, Ghana: an interrupted time series analysis

**DOI:** 10.1186/s41256-022-00271-1

**Published:** 2022-10-21

**Authors:** John Kanyiri Yambah, Kofi Akohene Mensah, Naasegnibe Kuunibe, Kindness Laar, Roger Ayimbillah Atinga, Millicent Ofori Boateng, Daniel Opoku, Wilm Quentin

**Affiliations:** 1 University Health Services, University of Education, Winneba, Ghana; 2 Department of Health Policy, Management and Economics, School of Public Health, Kwame Nkrumah University of Science and Technology, Kumasi, Ghana; 3 Department of Economics, Faculty of Social Science and Arts, SD-Dombo University of Business and Integrated Development Studies, Wa, Ghana; 4 Department of Public Administration and Health Services Management, University of Ghana Business School, Accra, Ghana; 5 Department of Community Health, Ensign Global College, Kpong, Ghana; 6 Department of Health Care Management, Technische Universitat Berlin, Berlin, Germany

**Keywords:** Capitation policy, Maternal care, Anemia tests, Delivery, Hospitals, CHPS

## Abstract

**Background:**

Payment methods are known to influence maternal care delivery in health systems. Ghana suspended a piloted capitation provider payment system after nearly five years of implementation. This study aimed to examine the effects of Ghana’s capitation policy on maternal health care provision as part of lesson learning and bridging this critical literature gap.

**Methods:**

We used secondary data in the District Health Information Management System-2 and an interrupted time series design to assess changes in level and trend in the provision of ANC4+ (visits of pregnant women making at least the fourth antenatal care attendance per month), HB36 (number of hemoglobin tests conducted for pregnant women who are at the 36th week of gestation) and vaginal delivery in capitated facilities-CHPS (Community-based Health Planning and Services) facilities and hospitals.

**Results:**

The results show that the capitation policy withdrawal was associated with a statistically significant trend increase in the provision of ANC4+ in hospitals (coefficient 70.99 *p* < 0. 001) but no effect in CHPS facilities. Also, the policy withdrawal resulted in contrasting effects in hospitals and CHPS in the trend of provision of Hb36; a statistically significant decline was observed in CHPS (coefficient − 7.01, *p* < 0.05) while that of hospitals showed a statistically significant trend increase (coefficient 32.87, *p* < 0.001). Finally, the policy withdrawal did not affect trends of vaginal delivery rates in both CHPS and hospitals.

**Conclusions:**

The capitation policy in Ghana appeared to have had a differential effect on the provision of maternal services in both CHPS and hospitals; repressing maternal care provision in hospitals and promoting adherence to anemia testing at term for pregnant women in CHPS facilities. Policy makers and stakeholders should consider the possible detrimental effects on maternal care provision and quality in the design and implementation of per capita primary care systems as they can potentially impact the achievement of SDG 3.

## Introduction

Global concerns for the need to improve maternal and neonatal health have led to many interventions to reduce maternal and neonatal mortality by 2030 [1]. Health systems have developed different payment systems to balance incentives for service provision with the need for cost control [2]. However, many of these payment system reforms have mixed results on service provision [2, 3] and maternal health services in particular [4, 5].

In many Sub-Saharan African countries, health insurance schemes have played a key role in facilitating access and use of maternal health services and promoting a significant reduction of the high maternal morbidity and mortality in the sub-region [6, 7]. Health insurance schemes have also positively impacted increased services utilisation by pregnant women in Senegal and Mali [7], skilled birth delivery in Rwanda [8], and utilisation of postnatal care in Mauritania [9]. In Ghana, several studies suggest that more women receive ANC4+ visits per pregnancy and are likely to have a skilled provider delivery if covered by the National Health Insurance Scheme (NHIS) [10–15]. What remains unclear is whether payment systems such as the capitation payment mechanisms used by health insurance schemes influence the provision of maternal care.

Capitation payment is a fixed amount of money paid to a provider for an individual’s healthcare needs over a specified period [16]. Blended capitation, where capitation is combined with another payment system for provider payment, have been used in several countries [17]. The impact of these capitation based payments on the quality and quantity of maternal health services have been mixed. Whereas some studies report an improvement [18–20], others suggest a worsening of the quality of prenatal care [21, 22]. In high income countries, capitation payment is common in primary care settings [23].

Capitation as a payment mechanism is uncommon in African health systems. Currently, many African countries are using capitation in combination with other payment methods [24, 25]. Kenya is a case in point where capitation was combined with Fee for Service for provider payment [24]. In Ghana, a capitation pilot was introduced for primary care in 2012 and discontinued in 2017 [26, 27]. In that pilot, the NHIS clients chose their preferred primary provider (PPP) to obtain needed outpatient health services with an option of a change of provider after 6 months [26]. Emergency care was to be paid for under the Ghana Diagnosis Related Groups (G-DRGs), while specialised services (referral) were to be paid for under the DRG. To qualify for reimbursement non-emergency referrals must emanate from a client's PPP [28]. Clients had to pay for the cost of self referred specialised services. Non emergency primary care services obtained from a facility other than a client's PPP had to be paid for out of pocket [28]. The healthcare facilities considered for choice as PPP spanned from CHPS, health centres, polyclinics and hospitals [10, 26, 29]. Maternal health services, including routine antenatal care consultations, laboratory tests (venereal disease research laboratory tests, urine dipstick tests, hemoglobin checks), delivery and postnatal care were initially fully part of the basket of services covered in capitation. Later skilled delivery was dropped from the basket in response to concerns of stakeholders in 2014 [28, 30]. After the suspension of the policy in 2017, primary care reverted to being paid for under the G-DRGs and the National Health Insurance Authority (NHIA) no longer required clients to have a PPP [31]. The monthly payments to facilities by the NHIS were also discontinued [31]. Some studies show that capitation in Ghana achieved cost containment, created competition and encouraged the efficient use of resources [32] and reduced outpatient utilisation [33]. However, knowledge and perception of the policy were poor [26]. In the specific case of maternal health services, as posited by Comfort et al. [34], payment mechanisms in any insurance scheme can potentially affect their provision in health facilities. One of the reasons for capitation in Ghana was to provide financial resources for primary outpatient service provision, especially those in the capitation basket [28]. The NHIS reimbursements were typically in arrears for up to 6 months; hence, capitation payments served as a critical resource for service providers and service provision since these were predictable and always paid on time [32]. Therefore, the subsequent withdrawal of the capitation policy could potentially affect service provision in general and critical maternal health services in particular. The uncentatinty about the likely effect of the piloted and its discontinuation on provision of maternal care represents a critical knowledge gap necessary for policy makers.

To fill this gap, we evaluated the effect of the withdrawal the capitation poliy on the provision of maternal care in CHPS and hospital level facilities. In this study we aimed to  analyse the levels and trends of provision of ANC4+, HB36 and facility delivery in CHPS and public hospital level facilities during and after the suspension of the capitation policy in the Ashanti Region of Ghana.

## Methods

### Study design

We used the single group interrupted time series design to estimate the effects of the withdrawal of the capitation policy by examining the change in level and slope of three maternal health service provision while controlling for secular trends[35]. This design is appropriate where no comparison group is available. The effects of the policy withdrawal are then deduced from the level and trend changes after the intervention [35].

### Study setting

The study was conducted in the Ashanti Region of Ghana, where capitation was introduced in January 2012 and discontinued in August 2017. The Ashanti region was selected for piloting the capitation to control costs arising from high expenditure on claims [33]. Even though the capitation covered all facilities, including CHPS, we focused on maternal care provison in the 720 CHPS facilities and the 25 primary district hospitals in Ashanti region. The focus on maternal health stems from the increased trend in inequality in the use of maternal health services in recent time in health facilities and the potential that these are affected by the capitation policy. We also focused on these two facility types (CHPS and hospitals) because they typically close the interaction between the start and end of primary maternal care provision. Additionally, CHPS form one of Ghana’s pathway to achieving Universal Health Coverage (UHC) through primary health care (PHC) [36]. In Ghana’s referral system, the polyclinic and health centres are supposed to receive referrals from CHPS facilities and in turn refer to the district hospital for all medical conditions they cannot treat [37]. However, in practice, CHPS and polyclinics often refer directly to the hospital in their catchment area [38].

### Data sources

Data were obtained from the DHIMS 2 which is an electronic database introduced to help healthcare managers improve upon the collation and analysis of routine service data [39]. The DHIMS 2 keeps database for services provided across all facilities. We extracted maternal health service provision data for all CHPS and public hospitals from the database. This database is managed by the Centre for Health Information Management (CHIM) of the Ghana Health Service. Every month, health facilities supply routine healthcare data for entry into this database [39].

### Data access and extraction

Data were extracted in Excel by a trained District health information officer between October 1, 2020 and November 31, 2020 using a data extraction sheet. The data were transferred from Excel to Stata 15.0 for management and analysis.

### Sample size

The sample comprised all the 720 CHPS and 25 primary district hospitals with data sets available in the DHIMS 2. The indicators of interest were aggregated on monthly basis across the 31 consecutive months preceding the withdrawal of capitation (from January 2015 to July 2017) and for 29 consecutive months after the withdrawal of the capitation, from August 2017 to December 2019.

### Participants/study population

The study participants constituted pregnant women who had recorded more than four antenatal visits and attended a health facility in a given month. It also included women who had their hemoglobin levels checked at their 36th week and mothers who delivered in a given month in CHPS facilities and hospitals.

### Study variables

The study included three maternal care indicators; ANC4+, HB36 and delivery. ANC4+ represents the number of women who received at least their fourth scheduled ANC visit in a particular month. HB36 represents the number of laboratory tests done for women in their 36th week of pregnancy in a given month. Delivery represents the number of births delivered at a facility in a given month. These indicators were chosen because, they are indices of provision of quality maternal care in any pregnancy. While ANC4+ measures the likelihood of receiving effective maternal health interventions [40], HB36 measures the health facility’s readiness in the assessment and preparation to identify and manage anemia in the peripartum period. Despite being dropped from the capitation basket, (vaginal) delivery rates in health facilities are surrogate measures of skilled birth uptake and also the acceptability of maternal care services by pregnant women.

### Statistical analysis

Summary statistics such as the mean, minimum, maximum and the standard deviation of maternal health service provision indicators during capitation and after its discontinuation, were calculated. The segmented regression model was employed to analyse the levels and trends of service provision during and after the discontinuation of capitation. The regression is presented in Eq. (1):

1 $$Yt = \, {\beta}0 \, + {\beta}1Tt \, + \, {\beta}2Xt \, + {\beta}3XtTt \, + et \ldots ,$$

where *Yt* is the maternal care indicator of interest, $\beta 0$ represents the intercept or pre-withdrawal level of care before the withdrawal of capitation, $\beta 1$ is the trend in the provision of care until the withdrawal of capitation, $\beta 2$ represents the change in the level care provision in theperiod immediately following the withdrawal of capitation (compared to the counterfactual), and $\beta 3$ represents the difference between pre-and post-capitation withdrawal trends in the provision of care.

Significant *P* values in $\beta 2$ show an immediate effect of the withdrawal of capitation and in $\beta 3$ show effects of capitation withdrawal until Dec 2019. Autocorrelation was corrected and the corresponding Dubin-Watson (DW) statistic was reported. DW values close to 2 indicate no serious autocorrelation. Statistical significance was set at 0.05.

## Results

### Descriptive statistics

The average number of monthly ANC4+ visits in CHPS level facilities increased from 253 (min: 165, max: 383, SD: 50) during capitation to 389 (min:304, max: 544 SD: 54.01) after the discontinuation of the capitation policy. However, in the hospital level, average monthly ANC4+ visits decreased from 2553 (min:1721, max:4579, SD:778) during the capitation period to 1953 (min:1462, max 2569, SD:273) post capitation withdrawal. The results are shown in Table 1.

**Table 1:** Average provision of maternal care

Period	During capitation				After withdrawal of capitation			
Indicator	Mean	SD	Min	Max	Mean	SD	Min	Max
ANC4+ CHPS	253.19	50.09	165	383	389.34	54.01	304	544
ANC4+ hospital	2553.35	778.36	1721	4579	1953.83	272.99	1462	2569
HB36 CHPS	249.16	130.78	63	577	191.86	54.14	112	378
HB36 hospital	1684.61	271.53	1232	2443	1493.64	179.98	1162	2037
Delivery CHPS	184.42	45.66	119	278	313.62	52.45	227	416
Delivery hospitals	2103.48	218.14	1736	2484	2084.72	195.83	1745	2495

*Source*: Authors’ calculations based on DHIMS 2, 2015–2019

When considering HB36 for CHPS level facilities, Table 1 shows that the average number of HB36 checks performed per month decreased from 249 (min: 63, max:577, SD: 131) during the capitation period to 192 (min:112, max 416; SD 52) after discontinuation. In hospital level, average monthly checks dropped from 1684 (min 1232, max 2443, SD 272) to 1494 (min:1162; max:2037, SD: 180) after discontinuation of capitation.

By contrast, the average number of deliveries increased from 184 (min 119; max: 278 SD: 46) during capitation to 314 (min: 227; max: 416; SD 52) for the period after the discontinuation of capitation in CHPS facilities, while in hospitals the average deliveries decreased from 2103 (min:1736, max:2484, SD:218) to 2085 (min:1745, max:2495, SD:196).

### Regression results

Table 2 shows that the capitation policy's discontinuation did not significantly affect the pre-existing level of care provision for all three indicators (ANC4+ visits, HB36 tests or deliveries) in CHPS or hospitals.

**Table 2:** Regression results of the effect of the withdrawal of capitation on maternal health care provision

Period	During capitation		After capitation withdrawal		D–W statistic	
Indicator	Level	Trend	Level change	Trend change	Original	transformed
CHPS ANC4+	188.18***	4.46***	− 11.20	− 0.65	1.68	2.05
Hospital ANC4+	4912.57***	− 63.61***	119.61	70.99***	1.49	1.90
CHPS HB36	182.53***	4.56**	− 90.43*	-− 7.01**	1.44	2.07
Hospital HB36	2738.78***	− 24.44***	23.27	32.87***	1.86	1.97
CHPS delivery	122.76***	4.26***	− 11.92	0.57	1.10	1.69
Hospital delivery	3168.03***	3.75	− 226.26	6.11	1.27	1.68

*Source*: Authors’ calculations based on DHIMS 2, 2015–2019

***p < 0.001; **p < 0.05; *p < 0.10

**Fig. 1:**
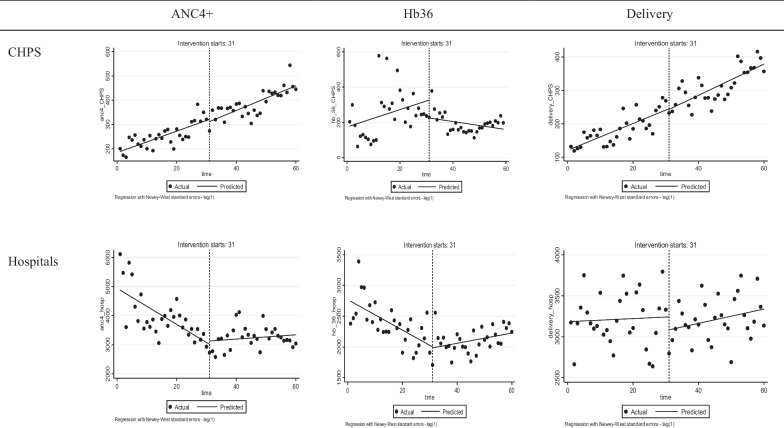
Interrupted time series graphs for ANC4+, HB36 and deliveries. *Source*: authors based on DHIMS 2

However, there were some effects on the trend of provision of care for these indicators. Specifically, we observed a statistically non significant increasing trend for ANC4+ visits in CHPS, which continued after the withdrawal of the policy. For hospitals, a declining trend during capitation (− 63.61), changed into a significantly positive trend after capitation (70.99) as shown in Table 2 and Fig. 1.

The results further show that in CHPS, an increasing trend in HB36 tests during the capitation policy was reversed after the discontinuation of the policy (− 7.01). By contrast, in hospitals, a declining trend in the number of HB36 during capitation, changed into an increasing trend after the end of capitation (32.78).

Concerning deliveries, the discontinuation of capitation had no significant effect on the trend in CHPS or hospitals.

## Discussion

This study evaluated the effect of the withdrawal of capitation on maternal healthcare indicators (ANC4+, HB36 and vaginal delivery) in CHPS and hospitals. The key finding was that the withdrawal of the capitation policy was followed by an increase in the trend of provision of ANC4+ and HB36 in hospitals while hindering the trend of HB36 at CHPS facilities. Other findings were that there was no effect of the withdrawal of capitation on the trend of vaginal delivery rates in CHPS or hospitals.

Several reasons could account for the key finding of increased trends of ANC4+ and HB36 in hospitals compared to CHPS after the withdrawal (meaning hospitals were more incentivised to provide quality antenatal care after the capitation withdrawal).

Foremost, the finding confirm, hospitals preferred the G-DRG over capitation as a payment method for their services as reported by an earlier study in the four Regions where capitation was later extended to [29]. However, while Andoh-Adjei et al. [29] investigated preference of payment methods by healthcare providers through a perception study, the current study gives additional detail on service provision which focuses on a providers’ preferred payment method. The results however conflict Andoh-Adjei et al.’s finding that that in the case of Ashanti Region, providers preferred capitation to DRG for primary maternal care service delivery.

Also, it is possible that the increase in the trend of both ANC4+ and HB36 in hospitals after the policy withdrawal indicates that capitation failed to meet the quality of maternal care expectation by pregnant women. We argue so because ANC4+, an index of the likelihood of receiving effective antenatal contact for the improvement of the quality of maternal care and one of the tracer indicators for SDG 3.8.1[40] declined during the capitation pilot, only recovering after the discontinuation. ANC4+ measures the minimum required contact between pregnant women and health care providers for safe antenatal care. Similarly, the increased number of HB36 tests confirms the increased trend of compliance with the WHO checklist of tests required for quality care in pregnancy in Ghana at the 36th week [41]. This is in spite of the fact that prior trends in provision might have been mitigated by the blend of capitation with other payment methods for hospitals during the capitation policy implementation [42, 43]. While our the study agrees with findings by Hennig-Schmidt et al.[44], it conflicts with that of Ponce et al., and van-Dijk et al. [45, 46], that capitation is associated with an improvement in adherence to quality standards. The results of these earlier studies [44–46] may differ from our results because of the specific capitation packages, provider profiles and disease conditions used to evaluate service quality. Additionally these were not conducted on maternal health services.

Furthermore, the statistically significant increase in the trends of ANC4+ and HB36, after the policy withdrawal, could mean that capitation induced poorer provider–client relationships in hospitals, congruent with findings in Thailand [47], Canada [48] and the USA [49]. However, this argument is contradicts the expectation that capitation would increase provider–client trust [50].This is because for the specific case of maternal health services, in the Ghanaian context, pregnant women prefer providers they can trust [51] and will patronise their services more. This probably explains the trends of maternal service provision observed after the policy withdrawal.

Additionally, the significantly lower HB36 trends in CHPS after the withdrawal of capitation could imply that CHPS level facilities had a critical support for their laboratories from the stable advance payments in capitation. This observation is similar to findings by Mills et al. [47]. However, these results are contrary to expectations after findings by Andoh-Adjei et al. [29], that there were no significant association between the CHPS level of care and provider preference of G-DRG under capitation, probably due to the low number of CHPS level providers in their study. Once the per capita payments were no longer being made after the policy withdrawal, in CHPS, pregnant women were now referred to obtain these tests at hospitals. This is perhaps reflects in the the increased provision of HB36 tests observed in hospitals. On the other hand, it could be confounded by moral hazard or supplier induced demand [52] found by earlier studies as attributes of increased laboratory service for insured clients in hospitals.

Moreover, the capitation policy promised the reinforcement of the gatekeeper system with more stringent referral requirements [28]. After its withdrawal, increased trends of ANC4+ and HB36 in hospitals may indirectly indicate its effectiveness at gatekeeping during the policy, driving healthcare provision to lower-level facilities (CHPS and health centres). Although CHPS, were not the only lower level facility, the HB36 trends in CHPS (increased) and hospitals (decreased) prior to the withdrawal of capitation may lend support to this assertion.

Finally, the increased trends in ANC4+ and HB36 in hospitals post capitation suspension, may reflect changes in general service readiness (GSR) of hospitals. GSR indexes the readiness of a health facility to provide care [53]. It may have been the case that the increased GSR in hospitals after the introduction of the NHIS [53], declined contrary to policy expectations during capitation [30]. Increased HB36 trend in hospitals after the policy withdrawal suggests capitation may have been associated with lower GSR during its implementation even though the NHIS is historically noted to have increased GSR after its introduction [53]. This finding also suggests that DRG primary care payments in hospitals may be associated with increased service readiness rather than capitation.This confirms concerns expressed by stakeholders that the introduction of capitation would probably affect healthcare providers negatively [27].

Our study has some limitations. Though it relied on readily available secondary DHIMS 2 data, such data is commonly subject to input errors, extraction errors and data discrepancies. The extraction errors were attenuated by making a second health information officer extract the maternal care data for comparison. We relied on data validation routinely done by facility heads for all data monthly to apply to our results. To strengthen future research results, primary collation of facility data will increase the internal validity of studies of a similar type, although studies [39] have confirmed the completeness and accuracy of the DHIMS 2 data, especially, with respect to maternal care indicators. Ideally, we should obtain all data points before the introduction of capitation, during the capitation and after the removal of the policy to allow for a more robust inferential analysis. However, the 60 months considered is far above the minimum of 9 data points before and after a policy change for interrupted time series analysis [54]. Our study was not insulated from the effects of other system-level confounders; this could have been minimised by introducing a control group and multiple interruptions in the time series analysis and a difference in difference analysis. However, at the time of this study, it was not possible to have access to the national database since doing so required many bureaucratic procedures and processes that could not be overcome within the limited time available for conducting this research. Future studies using the national dataset, allowing for the inclusion of control regions, will shed more light on the increasing potential of the capitation payment system for maternal care provision. The study relied on aggregated data which calls for cautious interpretation. The study did not look at the individual pregnant women socio demographic characteristics in its design. Though a qualitative focused group discussion with participants will address this, it will create another problem of recall bias.

## Conclusions

The capitation policy repressed trends of provision of quality care in hospitals and improved the compliance of CHPS with the WHO recommended laboratory care in pregnancy The discontinuation of the policy probably impacted negatively on the gatekeeper system with regards to the provision of quality maternal care in hospitals. These findings imply that policy makers, particularly, the NHIA, may have to redesign and redefine the basket of services for a future capitation based payments for primary maternal care. This will help Ghana achieve the SDG targets of reduced maternal deaths (SDG 3.1), neonatal care (SDG 3.2) and improved PHC.

## Data Availability

The datasets generated and analysed during the study are not publicly available due to the institutional policy of the CHIM of the Ghana Health Service but are available from thecorresponding author on reasonable request.

## Abbreviations

ANC4+: Number of facility antenatal care attendance of atleast 4 in the index pregnancy
CHPS: Community based health planning and services
CHIM: Centre for health information management
DHIMS: District Health Information Management System
DW: Dubin Watson
GSR: General Service Readiness
HB36: Number of hemoglobin tests at 36th week of gestation
NHIA: National Health Insurance Scheme
NHIS: National Health Insurance Scheme
OECD: Organisation for Economic Cooperatiion And Development
PHC: Primary Health Care
SDG: Sustainable Development Goal
WHO: World Health Organisation
